# An Exploration of Parent-Youth Agreement on Functional Impairment in Adolescents Utilizing Outpatient Mental Health Services

**DOI:** 10.3390/healthcare6030106

**Published:** 2018-08-30

**Authors:** May Yeh, Argero Zerr, Raymond La, Kristen McCabe

**Affiliations:** 1 Department of Psychology, San Diego State University, San Diego, CA 92182, USA; azerr@sdsu.edu (A.Z.); rla@sdsu.edu (R.L.); 2 Child and Adolescent Services Research Center, San Diego, CA 92123, USA; kmccabe@sandiego.edu; 3 Department of Psychiatry, University of California, La Jolla, San Diego, CA 92093, USA; 4 Department of Psychological Sciences, University of San Diego, San Diego, CA 92110, USA

**Keywords:** parent-youth agreement, functional impairment, mental health services, adolescents

## Abstract

Parent-youth agreement on the youth’s functional impairment may have important implications for mental health service utilization, assessment, therapy goal development, and treatment engagement for adolescents. The present study examines parent-youth agreement on their perceptions of youth functional impairment in a predominantly racial/ethnic minority sample of adolescents utilizing outpatient mental health services. Parent and youth functional impairment ratings were compared, and agreement was estimated in multiple ways. On average, parents indicated higher levels of youth functional impairment compared to youth in their overall scores, and when differences existed between parents and youth at the functioning domain and item level. Although there was similarity in the proportion of parents and youth who reported total impairment above the clinical cut-off, actual agreement between parent-youth pairs was only slight. There appeared to be substantial variation in agreement levels when identifying problems in functional impairment at the domain and item levels, and some areas of strong consensus were identified. These findings highlight the need to consider parent-youth agreement in perceptions of functional impairment and the complexities that may underlie this agreement.

## 1. Introduction

Premature termination rates for youth in mental health services are generally concerningly high [1,2], and stronger parent-youth agreement on treatment goals has been linked to a greater frequency of treatment visits [3]. However, low rates of agreement between parents and youth on target problems for treatment have been reported. For example, 63% of parent-youth dyads in a sample of children referred to outpatient clinics did not agree upon a single target problem, and when problems were broadened into general areas, more than one-third of the dyads still did not agree on any one problem category [4]. Given these high levels of disagreement upon target problems, therapists may be faced with the difficult decision of whether to focus their therapy efforts on the problems voiced by the parent or by the child [5]. The implications of disagreement may be even more important for adolescent clients, who may have a stronger role in attending sessions than younger preschool or elementary school-aged children.

Parent-youth discrepancies in target goals may exist for numerous reasons, including varying reports on the presence of symptoms and differential perceptions of whether or not the youth’s functioning is impaired. Multiple studies have examined parent-child differences in agreement upon the presence of psychopathology, and meta-analyses of these studies have generally reported low to moderate levels of agreement [6,7]. However, few studies have investigated parent-child agreement on whether or not the youth’s behavior is considered functionally impairing. These types of studies are important, as discrepancies in perceptions of the child’s ability to function in daily life may have a strong impact on motivation to seek treatment, the therapist’s development of diagnoses, the types of treatment goals that are selected, and the level of engagement that families may have in care.

Studies that examined parent-child concordance on youth functional impairment have yielded varied findings, which may be the result of differential samples and methods of assessing self-reported functional impairment. For example, in their examination of parent-youth agreement on functional impairment in a sample of adolescents in inpatient and outpatient services, Kramer and colleagues [8] assessed functional impairment based upon parent/youth ratings of the frequency of problem occurrence (never to always) or dichotomous endorsement of occurrence (e.g., of an arrest). In this study, agreement based upon kappa statistics ranged from low to fair, with a greater likelihood of parent report of impaired functioning as compared to youth in general. However, some differences arose across domains; specifically, parent report of impaired functioning was more likely in the areas of relationships, role performance, and prosocial activities, while youth report was somewhat more likely for areas of school consequences, legal consequences, and peer delinquency [8]. In a separate study, Bein et al. [9] investigated parent-child concordance on reports of distress, impairment, and symptoms for youths at an outpatient facility. Parents generally reported greater impairment as compared to their children on measures asking the amount of difficulty the child’s emotional/behavioral problems cause in the child’s ability in particular areas. Frank et al. [10] used what they describe as an “item to criterion” method for assessing functional impairment in a sample of adolescents in inpatient services and found that both relative agreement and absolute agreement between parents and youths were stronger for domains that were “public” as compared to “private”.

Other studies have investigated parent-youth agreement on functional impairment specific to particular diagnoses or histories. For example, in a sample of youth with a history of trauma, Oransky et al. [11] found no significant differences between caregivers and children in functional impairment as assessed by a post-traumatic stress disorder (PTSD)-focused measure. Keuthen et al. [12] reported that overall parent-child agreement was good on reports of functional impairment as a result of the child’s hair-pulling in a sample of youth with trichotillomania. Piacentini et al. [13] examined parent-child agreement on an obsessive-compulsive disorder (OCD)-specific measure in a sample of youth with obsessive-compulsive disorder and found that significant impairments in home functioning and school functioning were more likely to be reported by parents as compared to children.

Taken together, these studies highlight the variability of parent/youth-report functional impairment measures in terms of specificity to a diagnosis as well as the means for determining impairment (e.g., reports of frequency/occurrence of a problem or a respondent’s judgment about interference with functioning). As such, measures may have correspondingly varied results and serve different, important purposes, such as informing diagnoses, understanding impairment specific to a particular disorder, or developing treatment goals. When exploring reasons for differential agreement on target problems for therapy, it may be particularly salient to examine parent-youth agreement on the extent to which functioning across a broad range of areas is viewed as problematic from the respondent’s subjective perspective (e.g., both parent and youth may agree that the youth is failing out of school, but they may not both agree that this is a concern). If parents and youth agree that a particular area of the child’s functioning is problematic, this may form the basis for treatment goals that both parent and youth may be motivated to pursue.

The present study will build upon the current literature by examining parent-youth agreement on non-diagnostic-specific, respondent-rated functional impairment in a predominantly racial/ethnic minority sample of adolescents who were receiving outpatient mental health care. The study does not center upon a particular diagnosis or risk factor, and the focus will be upon a measure that asks parents and youth to report upon the extent to which they believe a particular area is problematic, rather than upon measures that ask about the frequency or occurrence of problems that may be evidence of functional impairment. The examination of concordance in a predominantly racial/ethnic minority sample is particularly important given the potential impact of culture on mental health-related constructs (e.g., racial/ethnic differences in adolescent reasons for receiving mental health care [14], racial/ethnic differences in parental etiological beliefs about the causes of child problems [15], and the role acculturative processes may play in problem identification [16]) and high treatment dropout rates for ethnic minority children [17,18]. The study will describe parent-youth agreement framed around two questions: (1) how similar are parent and youth reports of functional impairment; and (2) how much do parents and youth agree that various areas of functioning are problematic? Both of these questions will be explored at multiple levels of functional impairment: overall, domain, and item. We predict low parent-youth agreement on functional impairment as measured by this study, as parents and youth are different stakeholders with potentially differing frames of reference and contexts for judging the child’s behavior. However, given limited knowledge about parent-youth agreement on subjective ratings of functional impairment, this study’s analyses are considered exploratory.

## 2. Materials and Methods

### 2.1. Study Population

Participants were caregivers (hereafter called parents) and youth who took part in a longitudinal study of multi-stakeholder agreement on treatment-related constructs for youth who were receiving outpatient therapy. The study involved parent, youth, and therapist data collection related to 318 youth across 5 potential timepoints. The current analyses focus upon parent and youth Time 1 surveys (a description of participant recruitment and inclusion/exclusion criteria can be found in Yeh et al., 2016 [19]). Time 1 surveys involved in-person youth, parent, and therapist interviews that were conducted early in the treatment episode and included measures of demographics, explanatory model components, functioning, symptomatology, and other potential predictors of stakeholder cognitive match. Parent/youth interviews were completed in English or Spanish. Institutional Review Board approval and informed consent were obtained prior to interview completion (protocol number: 364060). Parents/youth received $30 in gift certificates/compensation for participating in the Time 1 interviews.

In order to try to focus upon agreement at earlier treatment stages, the current analyses excluded dyads if therapist interviews took place more than 5 sessions after the initial treatment session and parent/youth interviews occurred more than 30 days after the initial treatment session. On average, youth Time 1 interviews took place 18.03 days after the first treatment session (SD = 13.90) and parent Time 1 interviews took place 17.84 days after the first treatment session (SD = 13.26). Exclusions were also made due to clustering considerations (i.e., youths with a sibling also participating in the study, clinic with a single study participant), resulting in a sample of *n* = 285 dyads. Further exclusions were made for missing data; *n* = 8 cases were excluded if either the parent or the youth was missing the functional impairment measure at the Time 1 interview. The resultant subsample involved 277 parent-youth dyads.

### 2.2. Measures

Youth functional impairment was assessed by parent- and youth-report versions of the Columbia Impairment Scale [20]. The Columbia Impairment Scale (CIS) is a 13-item scale that reliably assesses the youth’s functional impairment across a broad range of areas. Parent and youth respondents in this study rated “how much of a problem” the respondent thinks the youth has with 13 items as: 0 = no problem, 1 = little problem, 2 = some problem, 3 = big problem, 4 = very big problem. Respondents were able to indicate that an item was not applicable (e.g., item related to getting along with the child’s father; these were rescored to 0 prior to analysis in this study to indicate no problem was noted in this area). Item scores were summed to create a total score of functional impairment. Bird et al. [20] recommend a total score of ≥15 as a cut-off for “definite impairment”. As per other studies [21,22,23], a total score of ≥15 will be used as a clinical/clinically-significant cut-off. Multiple studies have demonstrated that the CIS has strong psychometric properties as a unidimensional measure, including internal consistency, test-retest reliability, and construct and concurrent validity [20,24].

Domains of functional impairment for this study were based upon those identified by Singer et al. [25]. Singer et al. [25] conducted exploratory factor analyses using the parent version of the CIS with a sample of mothers seeking mental health services for their children. Three factors involving 12 items of the CIS (omitting the item that asks about getting along with the child’s father) were identified as follows: home/family (5 items), school (4 items), and socialization (5 items). Two items had cross-loadings onto two domains. Continuous domain scores involved summing the items of those domains. For the purposes of this study, problematic functional impairment in a domain was defined as one or more items in a domain with a rating of 3 (big problem) or 4 (very big problem).

Items were examined individually as continuous variables (0–4) and as dichotomous variables delineating problematic functioning (1 = problematic, 0 = not problematic). In this study, the respondent’s identification of problematic functioning at the item level was defined as a rating of either 3 (big problem) or 4 (very big problem) for that item.

### 2.3. Analytical Approach

Previous studies used both dichotomous variables [8,13] and continuous variables [9,10,11,12] to analyze agreement on functional impairment. However, to our knowledge, no study has analyzed parent-child agreement on functional impairment using the original CIS unidimensional measure or the subscales developed by Singer and others. Therefore, agreement was analyzed by using both dichotomous and continuous variables due to the exploratory nature of this study, to allow examination of agreement on both types of variables, and to facilitate comparisons to previous studies that utilized different measures.

The first set of analyses involved a series of paired-samples *t*-tests comparing parent and youth scores on CIS functional impairment in total, 3 domains (school/work, socializing and home/family), and 13 individual items.

The second set of analyses used a series of non-parametric tests to compare parents and youth on problematic levels of CIS functional impairment at the total, domain, and item levels. First, the proportion of parents and youth reporting problematic functional impairment at the total, domain, and item levels was compared using McNemar’s test statistic for paired dichotomous data. Second, dyadic agreement between parents and youth was assessed by calculating overall percent agreement (including agreement on both the presence and absence of a problem), percent agreement on problem endorsement, percent of youth agreeing with parent’s problem endorsement, and percent of parents agreeing with youth’s problem endorsement. In addition to calculating agreement via percentages, the strength of parent-child agreement was estimated using kappas and Guilford’s G statistics. Kappas are the most commonly used estimate of interrater agreement; however, given the limitations with kappas for skewed data, we also estimated agreement using Guilford’s G [26], which may provide more appropriate estimates of agreement for low base rate items [27]. Agreement using kappa and G levels were interpreted as follows: 0 to 0.20 slight agreement, 0.21 to 0.40 fair, 0.41 to 0.60 moderate, 0.61 to 0.80 substantial, and 0.81 to 1.00 almost perfect or perfect agreement [28].

## 3. Results

### 3.1. Participant Characteristics

Youths were aged 12 to 18 (M = 14.05 years, SD = 1.57) and were 40.1% female. Seventy-one point five percent of youths were Latino/Hispanic/Spanish of any race (hereafter referred to as Latino). In addition to those who were Latino, 13.7% of youths were African American/Black, 5.8% were non-Hispanic White, 1.8% were Asian American/Pacific Islander, and 0.4% were American Indian/Native American/Alaska Native, and 6.9% were Multiracial. All youth were utilizing outpatient mental health services; 78.3% of youths were utilizing school-based services, while 21.7% of youths received clinic-based services. Seven point nine percent of youths received multi-systemic therapy (MST).

Parents were aged 26 to 81 (M = 42.20 years, SD = 9.02), and 89.9% were female. Sixty-eight point four percent of parents were Latino. In addition to those who were Latino, 14.5% of parents were African American/Black, 9.1% were non-Hispanic White, 1.8% were Asian American/Pacific Islander, 0.4% were American Indian/Native American/Alaska Native, and 5.8% were Multiracial (0.7%, or *n* = 2 were missing racial/ethnic information). Mean household income was $21,915 (SD = $14,435), and highest educational levels were as follows: 28.2% through 8th grade, 13.0% up to grades 9–12 without a diploma, 27.4% obtained a high school diploma, GED, or other certification, 21.3% had some college education but did not obtain a degree, and 9.4% had a college degree or above. Two parents (0.7%) were missing education information.

### 3.2. Comparisons of Parent and Youth Reports of Functional Impairment

Parents reported significantly higher total functional impairment (M = 16.81, SD = 8.96) compared to youth (M = 15.49, SD = 8.15; *t* = 2.21, *df* = 268, *p* = 0.028). The average difference between parent and youth scores was 1.32 (SD = 9.82). There were 12 dyads (4.5%) where the parent and youth reported the exact same overall functional impairment. Of the remaining cases, 141 parents (52.4%) reported a higher overall score than youth, and 116 youth (43.1%) reported a higher overall score than their parent. Alternatively, there were 180 dyads (66.9%) where parents and youth were within 1 standard deviation (9.82) of each other, 59 dyads (21.9%) where parents were higher than youth, and 30 dyads (11.2%) where youth were higher than parents. Overall, parent and youth total functional impairment scores were moderately correlated (*r* = 0.34, *p* < 0.001).

At the domain level, parents and youth were significantly different on the socializing domain (*t* = 3.00, *df* = 273, *p* = 0.003), with parents reporting significantly higher functional impairment (M = 5.45, SD = 3.73) than youth (M = 4.67, SD = 3.46). Parents (M = 6.22, SD = 3.57) and youth (M = 6.09, SD = 3.33) were not significantly different on the school/work impairment domain (*t* = 0.53, *df* = 272, *p* = 0.595). There were also no significant differences between parents (M = 6.93, SD = 4.36) and youth (M = 6.58, SD = 3.78) on the home/family impairment domain (*t* = 1.29, *df* = 271, *p* = 0.199). Correlations between parent and youth domain scores were moderate to large: *r* = 0.29 for the socializing domain, *r* = 0.37 for the school/work domain, and *r* = 0.42 for the home/family domain (all *p*s < 0.001).

At the item level, parents and youth differed significantly on 6 of the 13 functional impairment items. Specifically, parents reported higher functional impairment than youth on: feeling unhappy/sad (*p* < 0.001), having fun (*p* = 0.030), feeling nervous or worried (*p* = 0.015), getting along with brothers/sisters (*p* = 0.003), schoolwork (doing his/her job) (*p* = 0.004), and behavior at home (*p* < 0.001). Youth reported marginally higher functional impairment compared to parents on one item pertaining to getting into trouble (*p* = 0.062). Parents and youth did not significantly differ on the remaining 6 functional impairment items: getting along with mother, getting along with father, behavior at school (or job), getting along with adults other than mother/father, getting along other kids, or activities like sports/hobbies (all *p*s > 0.05).

### 3.3. Assessment of Parent and Youth Agreement on Problematic Areas of Functioning

There were no significant differences between the proportion of parents (56.13%) and youth (55.02%) who reported that total functional impairment was above the clinical cutoff (McNemar’s test *Х*^2^ = 0.04, *df* = 1, *p* = 0.847). However, as shown in Table 1, there was only slight agreement between parents and youth on whether functional impairment reached clinical significance or not. Among parent-youth dyads who agreed on the clinical significance of functional impairment, it was more common for parents and youth to agree when parents and youth both rated functional impairment as above the cutoff (*n* = 96; 59.3%) compared to below the cutoff (*n* = 66; 40.7%). Among parent-child dyads who disagreed, rates of agreement were relatively similar for dyads in cases when parents reported above the clinical cutoff (*n* = 55; 51.4%) compared to when youth reported above the clinical cutoff (*n* = 52, 48.6%).

There were significant and marginally significant differences between the proportion of parents and youth who reported that functional impairment was problematic in certain domains. Specifically, parents (39.78%) were significantly more likely than youth (31.39%) to identify socializing as a domain with problematic functional impairment (McNemar *Х*^2^ = 4.61, *p* = 0.031). Parents (50.18%) were also marginally more likely than youth (42.86%) to identify school/work as a domain with problematic functional impairment (McNemar *Х*^2^ = 3.54, *p* = 0.059). However, there were no significant differences between the proportion of parents (45.59%) and youth (44.85%) who identified that the home/family domain showed problematic functional impairment (McNemar *Х*^2^ = 0.01, *p* = 0.925). Overall, 40.1% (*n* = 108) of parent-youth dyads co-endorsed at least one domain as problematic. Specifically, 18.2% of dyads co-endorsed a single domain as problematic, 16% co-endorsed two domains, and 5.9% co-endorsed all three domains as problematic. As shown in Table 1, parents and youth showed fair agreement on the school/work domain, slight to fair agreement on the socializing domain, and slight agreement on the home/family domain.

Overall, parents identified more items as being problematic in functional impairment (M = 2.28) compared to youth (M = 1.83; *t* = 2.72, *df* = 268, *p* = 0.007). Proportionally, parents were more likely than youth to identify functional impairment problems on 4 items: feeling unhappy/sad, behavior at school (or job), schoolwork, and behavior at home (McNemar *p*s < 0.05). Overall, 31.6% (*n* = 85) of parent-youth dyads co-endorsed at least one item as problematic. Specifically, 13.4% of dyads co-endorsed a single item as problematic, 10% co-endorsed two items, 3.3% three items, 2.6% four items, 0.7% 5 items, 1.1% 6 items, and 0.4% co-endorsed 7 items as problematic. As shown in Table 2, agreement between parents and youth on identifying problems with functional impairment ranged from 65.45% (getting into trouble) to 91.34% (having fun). According to kappa estimates of agreement, parents and youth showed slight to poor agreement. Using Guilford’s G estimates produced greater variability on item level agreement; parents and youth showed fair agreement for 3 items: getting into trouble, behavior at school (or job), and schoolwork (doing his/her job); moderate agreement for 4 items: feeling unhappy/sad, getting along with brothers/sisters, activities like sports/hobbies and behavior at home; substantial agreement for 5 items: getting along with mother, getting along with father, getting along with other adults, feeling nervous or worried, and getting along with other kids; and 1 item with almost perfect agreement: having fun.

## 4. Discussion

The present study examined parent-youth agreement on the youth’s functional impairment, as defined by ratings of the problematic nature of the child’s functioning in particular areas. Comparisons between parent and youth scores as well as calculations of agreement regarding the identification of problems in functioning were made. When differences existed, parent scores of youth functional impairment were higher than those of youths, and additional meaningful patterns of agreement and disagreement emerged upon further examination of agreement through multiple perspectives and methods.

The first part of the study focused upon parent-youth agreement by comparing the similarity between their ratings of functional impairment when examined as continuous variables. Parents reported higher total youth functional impairment as compared to their children. Further examination of the data yielded some additional interesting observations. The mean total functional impairment score was above the clinical cut-off for both youths and their parents. Parent and youth scores were moderately correlated, and the mean difference between parent and youth scores was small. Our findings indicate that while parent total scores were on average higher than those of youths, the majority of dyads (almost 70%) had scores that were within a standard deviation of one another. For dyads whose scores were separated by more than a standard deviation, twice as many parents had overall impairment scores that were higher than those of their children, as compared to youths who had scores higher than those of their parents.

When parent-youth comparisons were made according to the domains developed by Singer et al. [25], parents reported greater impairment in socializing as compared to their children, but there were no significant differences in either school or home/family domains. Correlations between parents and youth were moderate to large across the three domains. Parents reported significantly greater impairment on just under half of the items as compared to youth, including two internalizing symptoms: feeling unhappy/sad and feeling nervous or worried. There were no items for which youth were significantly higher than parents. Interestingly, the items for which parents possessed higher ratings did not form a consistent pattern across setting: parent ratings were higher than child ratings for schoolwork (doing his/her job) but not for behavior at school (or job); higher for behavior at home, but not for getting along with mother or getting along with father; and higher for having fun, but not for activities like sports/hobbies.

When turning to the question of whether or not parents and youth agree upon whether or not functional impairment reaches the threshold for being problematic, the extent of parent-youth agreement depended upon the metric used for measurement. There was no significant difference between the proportion of parents and youths whose total impairment scores were over the clinical cut-off, but when looking into actual dyadic concordance, kappa and G statistics indicate only slight agreement in this area. Taken together, these findings suggest that while a similar proportion of parents and youth in this outpatient therapy sample may rate the youth’s functioning as significantly impaired overall, the agreement between actual parent-child pairs is still very low. For those parent-youth dyads whose scores were both either above or below the clinical cutoff, almost 60% showed this agreement for being above the cut-off, as compared to just under 40% agreeing on scores that were below the cut-off. In contrast, dyads that were not in agreement related to the clinical cut-off were nearly equally split between parent or youth score being above the cut-off.

When examining whether parents and youth agreed that at least one item of a domain was problematic, findings roughly mirrored those of the continuous variables. There was a higher proportion of parents who identified at least one item in the socializing domain as problematic compared to youth, with either marginal or no significant differences between parents and youth in the school/work or home/family domains. Agreement as assessed by kappa and G statistics yielded slight to fair agreement across domains. When looking at agreement upon the presence of at least one problematic item in a domain (i.e., parent and youth both indicated that at least one item in that domain was in the problematic range, or parent-youth agreed that no problems existed for any item in that domain), agreement ranged from about 58% to 63%. This meant that for each of the domains, about 40% of the dyads completely disagreed about whether or not a problem existed in that particular domain. Not surprisingly, when focusing upon whether or not a definite problem exists for an item within a domain, the rates of agreement for the three domains were all below 30%. When parents identified a problematic item within a domain, youth agreement with the parent ranged from 41% to 55%. When youth identified at least one problematic item within a domain, parent agreement with the child ranged from 52% to 65%. These results indicate that, although some level of agreement exists between parents and youth, rates of agreement are still concerning for those areas that are identified by at least parent or child as being problematic, even in a sample of youth who were receiving mental health services.

At the item level, parents rated more items as problematic compared to youth overall, and their ratings were higher on four items that were again, across the spectrum of settings: feeling unhappy/sad, behavior at school (or job), schoolwork, and behavior at home. Dyadic agreement as interpreted using Landis and Koch [28] ranged from slight to fair agreement as per kappas, and ranged from fair to almost perfect with Guilford’s G estimates. The range of parents and youth who agreed as to the existence or non-existence of definite impairment on an item ranged from 65.45% to 91.34%. Interestingly, the poorest percent agreement was found on getting into trouble, behavior at school, and schoolwork, which are items that may be more likely to have more objective measures (e.g., grades, detention). In contrast, items pertaining to getting along with mother, father and other adults were associated with consistently better rates of agreement between parents and youth. Taken together, the findings suggest that overall, parents and youth have a high level of disagreement upon the degree to which the child’s functioning is problematic, but there are reasons to believe that their agreement may, in some areas, be quite strong. However, the results also point to the importance of considering not only whether there is agreement in general, but also about whether or not there is agreement when one stakeholder believes a problem exists. For example, fewer than one in three parents and youth co-endorsed at least one specific item out of the thirteen on the Columbia Impairment Scale as being a big or very big problem, and when expanded to domain agreement, the proportion only increased to about four out of ten. This is all the more striking, given a service-using sample whose families are presumably in some degree of distress, and the fact that most parents and youth endorsed at least one item as problematic.

This study extends previous work in the area by increasing the cultural diversity of the samples in which these constructs have been examined. Although prior studies utilized different methodologies for examining parent-youth agreement on functional impairment, some meaningful observations may be possible when comparing the present study to the existing literature. For example, our finding that parents reported higher total youth functional impairment as compared to their children was consistent with Bein et al. [9]’s report from a clinic that primarily served Caucasians. However, Frank et al. [10] found stronger agreement between parents and youth on “public” domains as compared to “private” while no clear pattern of agreement was apparent across setting in the present study. In addition, while, consistent with the previous literature, there were findings of low parent-youth agreement in general, the present study also found some instances of high agreement. These observations suggest that further extension of work into culturally diverse samples and the study of potential cultural influences upon parent-youth agreement on functional impairment may be warranted.

Facilitating co-endorsement on problematic areas of functional impairment may be particularly important when formulating target problems or treatment goals for youth therapy, as parent and youth perceptions of whether or not a functional impairment problem exists may greatly influence treatment engagement, adherence, and service use. If parent and youth both agree that the youth’s participation in daily life activities is compromised, they may both be more motivated for, engaged in, and committed to receiving services. Conversely, if either parent or youth disagrees that the youth’s functioning is impaired, motivation, engagement, and commitment may be negatively affected. The accuracy of diagnostic assessments may be impacted as well, particularly to the degree to which diagnostic determination is impacted by functional impairment. Future studies should examine if and how parent-youth agreement on functional impairment relates to diagnostic assessments, treatment engagement, and treatment outcomes. In addition, the field may benefit from greater exploration of the reasons for parent-adolescent disagreement on youth functional impairment, including the potential impact of generation, culture, acculturation level, perceptions about the presence of youth psychopathology, levels of insight, degree of parental monitoring, and the respondent’s own level of anxiety.

Several limitations to the study should be kept in mind. First, the current analyses did not take the youth’s symptomatology, treatment type, or length in therapy into account. Parent-youth agreement may have differed based upon the nature of the youth’s problems, service setting (e.g., school-based vs. clinic-based), and the type of treatment that the youth was receiving (e.g., multi-systemic therapy). In addition, although we tried to limit our sample to the early stages of therapy, there was still variability in the amount of time between the start of treatment and the first interview, which may have influenced parent-youth agreement, as it is possible that therapists may have spent time facilitating agreement in this way. Second, there are many ways of measuring agreement, and while we included several of them, we did not include all possibilities. The multiple methods that were used here may each be useful for different reasons.

In summary, this study adds to the literature with an examination of parent-youth similarity and agreement on the extent to which the youth’s functioning was problematic in a predominantly racial/ethnic minority sample of youth receiving outpatient mental health services. Our investigation focused upon parent-youth agreement on subjective ratings of functional impairment, which may be particularly important when considering reasons for discrepant treatment goals or target problems for therapy. Neither the sample nor the measure of functional impairment was disorder-specific. In addition, this study involved predominantly racial/ethnic minority sample on the West Coast that included a large proportion of Latinos, adding to the cultural diversity of the samples in which this topic has been studied. Furthermore, agreement was examined in multiple ways, yielding rich findings. Although, on average, parents and youth reported similar rates of overall clinical impairment when the sample was examined proportionally, actual dyadic agreement was quite poor. At the domain level, parent-youth agreement on impairment problems was slight to fair. Interestingly, there appeared to be substantial variation in parent-youth agreement on problematic functioning at the item level when examining concordance using Guilford’s G statistics (which may be more appropriate estimates of agreement for low base rate items) with agreement ranging from fair to almost perfect levels. Our findings suggest that parent-youth agreement is complex, and that there is utility in considering multiple ways of looking at this construct.

These results support the importance of understanding and facilitating agreement between youths and parents on functional impairment. The findings suggest that, while disagreement on the presence of functioning problems may be high overall, it is possible that some common ground between parents and youths may be found. Treatment providers are encouraged to consider eliciting both parent and youth perspectives on the youth’s functional impairment in order to inform treatment goal development as well as diagnosis. Previous work has shown a relationship between stronger parent-youth treatment goal agreement treatment and visit frequency [3]; it is possible that stronger agreement on areas of functional impairment may impact agreement on treatment goals and treatment engagement. It is recommended that future research investigate variables such as symptomatology, culture, acculturation levels, therapy type, and therapy/therapist characteristics that may influence agreement, therapist agreement with parents and youth on functional impairment, and the role that multi-stakeholder agreement may have on treatment engagement and outcomes.

## Figures and Tables

**Table 1:** Parent and youth agreement on problematic functional impairment total and domains.

Area	% Total Agreement (Whether Problem Exists or Not)	% Both Parent and Youth Endorse as a Problem	% Youth Who Agree with Parent That a Problem Exists	% Parents Who Agree with Youth That a Problem Exists	% Youth Who Agree with Parent That a Problem Does NOT Exist	% Parents Who Agree with Youth That a Problem Does NOT Exist	κ Agreement	G Agreement
Total Functional Impairment	60.22%	35.69%	63.58%	64.86%	55.93%	54.55%	0.20	0.20
School/Work Domain	62.64%	27.84%	55.47%	64.96%	69.85%	60.90%	0.25	0.25
Socializing Domain	61.68%	16.42%	41.28%	52.33%	75.15%	65.96%	0.17	0.23
Home/Family Domain	58.09%	24.26%	53.23%	54.10%	62.16%	61.33%	0.15	0.16

**Table 2:** Parent and youth agreement on problematic functional impairment items.

Item	% Total Agreement (Whether Problem Exists or Not)	% Both Parent and Youth Endorse as a Problem	% Youth Who Agree with Parent That a Problem Exists	% Parents Who Agree with Youth That a Problem Exists	% Youth Who Agree with Parent That a Problem Does NOT Exist	% Parents Who Agree with Youth That a Problem Does NOT Exist	κ Agreement	G Agreement
1. Getting into trouble	65.45%	8.00%	32.84%	30.56%	75.96%	77.83%	0.09	0.31
2. Getting along with mother	83.03%	5.05%	34.15%	41.18%	91.53%	88.89%	0.28	0.66
3. Getting along with father	85.45%	5.09%	43.75%	38.89%	90.95%	92.47%	0.33	0.71
4. Feeling unhappy/sad	74.18%	4.73%	20.31%	39.39%	90.52%	78.93%	0.13	0.48
5. Behavior at school (or job)	66.43%	9.39%	31.33%	41.94%	81.44%	73.49%	0.14	0.33
6. Having fun	91.34%	0.36%	6.67%	9.09%	96.18%	94.74%	0.03	0.83
7. Getting along with other adults	87.68%	1.81%	25.00%	20.83%	92.58%	94.05%	0.16	0.75
8. Feeling nervous or worried	80.36%	3.64%	23.26%	32.26%	90.95%	86.48%	0.16	0.61
9. Getting along with brothers/sisters	78.99%	7.61%	37.50%	47.73%	89.55%	84.91%	0.29	0.58
10. Getting along with other kids	86.18%	1.09%	15.79%	12.00%	91.41%	93.60%	0.06	0.72
11. Activities like sports/hobbies	79.78%	3.25%	21.95%	27.27%	89.83%	86.89%	0.13	0.60
12. Schoolwork (doing his/her job)	68.23%	9.75%	32.53%	45.76%	83.51%	74.31%	0.18	0.36
13. Behavior at home	77.54%	7.25%	32.79%	48.78%	90.23%	82.55%	0.26	0.55
